# Benefit-harm analysis and charts for individualized and preference-sensitive prevention: example of low dose aspirin for primary prevention of cardiovascular disease and cancer

**DOI:** 10.1186/s12916-015-0493-2

**Published:** 2015-10-01

**Authors:** Milo A. Puhan, Tsung Yu, Inge Stegeman, Ravi Varadhan, Sonal Singh, Cynthia M. Boyd

**Affiliations:** Department of Epidemiology; Epidemiology, Biostatistics & Prevention Institute, University of Zurich, Hirschengraben 84, Room HRS G29, CH-8001 Zurich, Switzerland; Department of Epidemiology, Johns Hopkins Bloomberg School of Public Health, Baltimore, USA; Department of Otorhinolaryngology – Head and Neck Surgery, University Medical Center Utrecht, Utrecht, The Netherlands; Brain Center Rudolf Magnus, University Medical Center Utrecht, Utrecht, The Netherlands; Department of Biostatistics, Johns Hopkins University Bloomberg School of Public Health, Baltimore, USA; Division of Biostatistics and Bioinformatics, Department of Oncology, Sidney Kimmel Comprehensive Cancer Center, Johns Hopkins University, Baltimore, USA; Division of General Internal Medicine, Johns Hopkins School of Medicine, Baltimore, USA; Center on Aging and Health, Division of Geriatric Medicine and Gerontology, Johns Hopkins School of Medicine, Baltimore, USA

**Keywords:** Prevention, Personalized, Cardiovascular, Cancer, Benefit-risk, Aspirin, Harm, Randomized trials, Guidelines

## Abstract

**Background:**

Clinical practice guidelines provide separate recommendations for different diseases that may be prevented or treated by the same intervention. Also, they commonly provide recommendations for entire populations but not for individuals. To address these two limitations, our aim was to conduct benefit-harm analyses for a wide range of individuals using the example of low dose aspirin for primary prevention of cardiovascular disease and cancer and to develop Benefit-Harm Charts that show the overall benefit-harm balance for individuals.

**Methods:**

We used quantitative benefit-harm modeling that included 16 outcomes to estimate the probability that low dose aspirin provides more benefits than harms for a wide range of men and women between 45 and 84 years of age and without a previous myocardial infarction, severe ischemic stroke, or cancer. We repeated the quantitative benefit-harm modeling for different combinations of age, sex, and outcome risks for severe ischemic and hemorrhagic stroke, myocardial infarction, cancers, and severe gastrointestinal bleeds. The analyses considered weights for the outcomes, statistical uncertainty of the effects of aspirin, and death as a competing risk. We constructed Benefit-Harm Charts that show the benefit-harm balance for different combinations of outcome risks.

**Results:**

The Benefit-Harm Charts (http://www.benefit-harm-balance.com) we have created show that the benefit-harm balance differs largely across a primary prevention population. Low dose aspirin is likely to provide more benefits than harms in men, elderly people, and in those at low risk for severe gastrointestinal bleeds. Individual preferences have a major impact on the benefit-harm balance. If, for example, it is a high priority for individuals to prevent stroke and severe cancers while severe gastrointestinal bleeds are deemed to be of little importance, the benefit-harm balance is likely to favor low dose aspirin for most individuals. Instead, if severe gastrointestinal bleeds are judged to be similarly important compared to the benefit outcomes, low dose aspirin is unlikely to provide more benefits than harms.

**Conclusions:**

Benefit-Harm Charts support individualized benefit-harm assessments and decision making. Similarly, individualized benefit-harm assessments may allow guideline developers to issue more finely granulated recommendations that reduce the risk of over- and underuse of interventions. The example of low dose aspirin for primary prevention of cardiovascular disease and cancer shows that it may be time for guideline developers to provide combined recommendations for different diseases that may be prevented or treated by the same intervention.

## Background

Consider a 60-year-old woman who comes to her general practitioner’s office and asks if she should take a low dose aspirin to prevent myocardial infarction (MI) or cancer. She has never had a cardiovascular event or cancer before, eats vegetables regularly, and is physically active. But she is a smoker, takes antihypertensive medication (current systolic blood pressure of 145 mmHg), has total cholesterol of 170 mg/dL, and HDL cholesterol of 55 mg/dL. The general practitioner determines the 10-year risks for both an MI [1] and stroke to be around 6 % [2] and for colorectal cancer to be around 2 % [3]. Uncertain about what to recommend to the patient, the general practitioner consults some guidelines on primary prevention of cardiovascular disease (CVD) and cancer.

Table 1 shows that the World Health Organization advises against low dose aspirin if the 10-year risk of an MI is below 30 % [4], the European Society of Cardiology [5] advises against low dose aspirin for all individuals, while the United States Preventive Services Task Force (USPSTF) [6] recommends aspirin if the risk is ≥8 % in women, because the benefit of lowering the risk of stroke outweighs the risk of major gastrointestinal (GI) bleeds. To date, the USPSTF recommends against aspirin for the prevention of colorectal cancer [7].

**Table 1:** Selected recommendations on low dose aspirin for primary prevention of CVD and colorectal cancer

Disease outcome	Organization	Recommendation and assumptions made
Cardiovascular disease	European Society of Cardiology [5]	Aspirin or clopidogrel cannot be recommended in individuals without cardiovascular or cerebrovascular disease due to the increased risk of major bleeding. (Class of recommendation III = is not recommended; level of evidence weak)
		No details on assumptions or on how benefit-harm assessment was done
Cardiovascular disease	World Health Organization [4]	− Coronary heart disease 10-year risk <10 %. For individuals in this risk category, the harm caused by aspirin treatment outweighs the benefits. Aspirin should not be given to individuals in this low-risk category. (1++, A)
		− Coronary heart disease 10-year risk 10 to <20 %. For individuals in this risk category, the benefits of aspirin treatment are balanced by the harm caused. Aspirin should not be given to individuals in this risk category. (1++, A)
		− Coronary heart disease 10-year risk 20 to <30 %. For individuals in this risk category, the balance of benefits and harm from aspirin treatment is not clear. Aspirin should probably not be given to individuals in this risk category. (1++, A)
		− Coronary heart disease 10-year risk ≥30 %. Individuals in this risk category should be given low dose aspirin. (1++, A)
		No details on assumptions or on how benefit-harm assessment was done.
Cardiovascular disease	US Preventive Services Task Force [6]	Encourage men age 45 to 79 years to use aspirin when the potential benefit of a reduction in MI outweighs the potential harm of an increase in gastrointestinal hemorrhage. (“A” recommendation)
		The reduction in MI outweighs the potential harm of an increase in gastrointestinal hemorrhage:
		− 45–59 year old if ≥4 % 10-year risk of coronary heart disease
		− 60–69 year old if ≥9 % 10-year risk of coronary heart disease
		− 70–79 year old if ≥12 % 10-year risk of coronary heart disease
		Assumptions for benefit-harm assessment:
		− Equal weights given to coronary heart disease, gastrointestinal hemorrhage, and hemorrhagic stroke
		− 10-year risk for coronary heart disease considered from 0–20 %
		− Average risk per age category considered gastrointestinal hemorrhage and hemorrhagic stroke
		− Ischemic stroke not considered
		− No competing risks considered
Colorectal cancer	US Preventive Services Task Force [7]	The USPSTF recommends against the routine use of aspirin and nonsteroidal anti-inflammatory drugs to prevent colorectal cancer in individuals at average risk for colorectal cancer.

This vignette illustrates the challenge of making (preventive) treatment recommendations for individuals and the disagreement among guidelines. In recent years, advancements were made in the development of evidence-based clinical practice guidelines. Major guideline developers and groups like the USPSTF or the Grading of Recommendations Assessment, Development and Evaluation (GRADE) Working Group strongly advocate to first systematically review the evidence on the benefits and harms of interventions and, in a second and separate step, to develop recommendations in a transparent way [8, 9]. Nevertheless, even if guidelines are based on high-quality systematic reviews and meta-analyses and follow GRADE or similar guidance, clinical practice guidelines and regulatory agencies sometimes disagree, as the example of low dose aspirin for cardiovascular primary prevention shows (Table 1) [6, 10, 11].

There are reasons for such disagreement among guidelines. Although many guidelines are now based on systematic reviews, few guideline developers use quantitative methods to estimate the benefit-harm balance of interventions [12, 13]. Also, drawing conclusions and issuing population-level recommendations just based on systematic reviews are problematic for some clinical questions, such as aspirin for cardiovascular primary prevention, because population-level recommendations are based on average outcome risks and do not reflect preferences of individuals [14–16], which may lead to over- and undertreatment [17, 18]. This may contribute to the generally rather low adherence to guidelines, along with other factors that make the transfer of knowledge into practice challenging [19, 20].

Compared to guidelines that make recommendations for entire populations, would it not be preferable to have treatment recommendations that are individualized through a patient’s own risks and preferences? And would it not be preferable to have more holistic guidelines on preventative measures that have an impact on more than one disease?

Typically, recommendations are organized separately by disease or medical specialty, and the USPSTF is currently developing or updating its three (separate) guidelines on aspirin for primary prevention of cardiovascular, disease, colorectal cancer, and other cancers [21]. Some have already argued that it may be time to move to guidelines that span across different diseases, which may be prevented by a common preventative measure such as aspirin [22]. To address these two limitations, our aim was to conduct benefit-harm analyses for a wide range of individuals using the example of low dose aspirin for primary prevention of CVD and cancer and to develop Benefit-Harm Charts that show the overall benefit-harm balance for individuals.

## Methods

### Selection of approach for benefit and harm assessment

We outlined a selection process for choosing a particular approach for a quantitative assessment of benefits and harms to illustrate benefit-harm balance in our previous work [23–25]. For this study, we selected the Gail/National Cancer Institute (NCI) approach as the multidimensional approach because it can consider risks of multiple benefit and harm outcomes, treatment effects on these risks, competing risks, and importance of outcomes (determined by patient outcome preferences, and when not available, by survival rates (for example, 5-year survival) and it summarizes all information in a number (benefit-harm index) [17]. This approach also provides a probability of net benefit, which captures major sources of informational uncertainty (see also the section on statistical analysis). In prior work [26], we compared this approach to the number needed to treat (NNT) approach, which is a well-known and established approach, and showed the Gail/NCI approach’s superiority in situations where there is more than an average benefit and an average harm outcome and where there are competing risks (such as death from other causes).

### Selection of data sources

For a quantitative benefit-harm assessment, several sources of evidence on treatment effects, risks of outcomes, and preferences for these outcomes are needed. We selected the following data sources:

a) Treatment effects: We used a recently updated meta-analysis of nine RCTs for the effect estimates of aspirin for primary prevention on the outcomes of MI and severe ischemic stroke (benefit outcomes) and on severe hemorrhagic stroke and severe GI bleeds [14]. The pooled relative risk for aspirin versus placebo was 0.86 (95 % confidence interval 0.74,1.00) for MI, 0.87 (0.73,1.02) for severe ischemic stroke, 1.35 (1.01,1.81) for severe hemorrhagic stroke, and 1.62 (1.31,2.00) for severe GI bleeds. We assumed a homogeneous treatment effect across the male and female populations since current evidence does not suggest heterogeneity of (relative) treatment effects [14]. A previous meta-analysis by the same authors formed the basis of recommendations of the most recent USPSTF statement on the benefits and harms of aspirin for cardiovascular primary prevention [6, 27].

For cancers, we used the summary measures of a meta-analysis of RCTs that assessed the effect of aspirin on long-term cancer incidence [28]. The pooled relative risk for aspirin versus placebo was 0.58 (0.44,0.78) for colorectal cancer, 0.55 (0.23,1.34) for biliary cancer, 0.51 (0.31,0.83) for esophageal cancer, 0.77 (0.49,1.22) for gastric cancer, 1.17 (0.50,2.71) for breast cancer, 0.84 (0.69,1.03) for lung cancer, 0.77 (0.59,1.01) for prostate cancer, 0.92 (0.66,1.29) for hematological cancer, 0.91 (0.59,1.40) for pancreatic cancer, 0.91 (0.54,1.51) for bladder cancer, 1.04 (0.40,2.73) for gynecological cancer, and 0.88 (0.48,1.61) for renal cancer. Some of the 95 % confidence intervals were wide and crossed 1.0 and the statistical models considered such statistical uncertainty.

b) Outcome risks: We stratified the analyses for men and women and four age categories since these two variables have a substantial effect on the risks of the outcomes and on all-cause death as a competing risk. We considered five risk categories both for MI and severe GI bleeds while we used age- and sex-specific outcome risks (that is, only one risk per age and sex category) for all other outcomes (severe ischemic stroke [29], hemorrhagic stroke [30], 12 different cancers [31], and death [32]). For each of the resulting combinations of outcome risks (2_sex_ *4_age_ *5_MI_ *5_gastrointestinal bleeds_ = 200) we repeated the benefit-harm analyses.

c) Weights for outcomes (importance): We searched for studies that elicited patient preferences for the outcomes using PubMed and the terms “patient preference”, “aspirin”, and “cost-benefit analysis”, as well as the “related articles” function. We identified one study that measured the preferences of participants that had not experienced cardiovascular events (primary prevention population) [15]. We also searched the Cost Effectiveness Analysis Registry of Tufts University [33] but did not identify additional studies from a primary prevention population. However, we did not find any preference-eliciting surveys on both cardiovascular and cancer outcomes, which would inform us about the relative importance of cardiovascular and cancer outcomes as needed for our analyses. Instead, and as an anchor for individual preferences, we used the 5-year survival rates of each of the outcomes and assigned a weight of 1.0 for outcomes with a 5-year survival rate of <50 %, 0.5 for those with 5-year survival rates of 50–90 %, and 0.1 for outcomes with a 5-year survival rate of >90 % (Table 2 [34–37]). We must emphasize that there is a wide variability of how people perceive the importance of outcomes. This is why we developed a website [http://www.benefit-harm-balance.com] where we illustrate how much different combinations of weights (according to individual outcome preferences) affect the benefit-harm balance.

**Table 2:** Weights assigned to outcomes included in the benefit-harm analyses of low dose aspirin for primary prevention of cardiovascular disease and colorectal cancer

Outcome	5-year survival	Weight
Myocardial infarction [34]	79.2 %	0.5
Ischemic stroke [35]	42.0 %	1.0
Hemorrhagic stroke [35]	42.0 %	1.0
Gastrointestinal bleeds [36]	45.5 % (3-year)	1.0
Colorectal cancer [37]	65.2 %	0.5
Biliary cancer [37]	18.3 %	1.0
Esophageal cancer [37]	18.3 %	1.0
Gastric cancer [37]	30.3 %	1.0
Breast cancer [37]	89.6 %	0.5
Lung cancer [37]	18.0 %	1.0
Prostate cancer [37]	98.9 %	0.1
Hematological cancer [37]	48.5 –85.8 %	0.5
Pancreatic cancer [37]	7.9 %	1.0
Bladder cancer [37]	77.4 %	0.5
Gynecological cancer [37]	45.8 –81.6 %	0.5
Renal cancer [37]	73.6 %	0.5

These weights can be adjusted individually on [http://www.benefit-harm-balance.com]

### Statistical analysis

For each of the combinations of outcome risks we used the Gail/NCI approach to estimate the benefit-harm balance. We first calculated the number of expected events (N) without aspirin for each of the 16 outcomes per 10,000 subjects over 10 years based on an exponential model that assumed a constant hazard rate over 10 years and adjusted for competing risks (here all-cause death). We then calculated the corresponding number of events with aspirin prevention, taking into account the treatment effects of aspirin from the meta-analysis. We calculated the difference in events between individuals with and without taking aspirin as an absolute measure of the preventive effect. We then calculated the benefit-harm index as the sum of differences in events for each outcome using the weights described above. To take into consideration the statistical uncertainty of the estimates for the treatment effects of aspirin, we performed simulations with 100,000 repetitions where we modeled treatment effects (log relative risk) using a normal distribution and obtained the probability that aspirin is beneficial as the proportion of index estimates (out of the 100,000 repetitions) that are positive [17, 38]. We assumed that outcome risks for MI and severe GI bleeds follow a uniform distribution within each risk category. We did not consider that aspirin may be discontinued after the occurrence of a bleed. As explained above, we repeated this analysis 200 times (that is, 200 simulations with 100,000 repetitions each) in order to get estimates of the benefit-harm balance for each of the 200 combinations of risk profiles. The simulations were done using R statistical software version 3.0.1 [39], and the codes are available on request. It took 25 s to run one simulation (100,000 repetitions for one combination of outcome risks) on a standard laptop (HP EliteBook 840).

### Construction of Benefit-Harm Charts

Similar to cardiovascular risk prediction charts, we constructed, for men and women separately, charts that inform individuals directly about the benefit-harm balance of aspirin [http://www.benefit-harm-balance.com]. We converted the probabilities that aspirin is net-beneficial for each of the combinations of outcome risks into a traffic light scheme. For results where ≥60 % of the 100,000 repetitions yielded a positive index (indicating that aspirin has a positive benefit-harm balance), we used the color green to indicate that the benefits of aspirin are likely to outweigh the harms. Accordingly, we used orange for results where 40—60 % of the 100,000 repetitions yielded a positive index (to indicate some uncertainty because the index is around 0) and red for results where <40 % of the 100,000 repetitions yielded a positive index (indicating that the benefit-harm balance is likely negative). The benefit-harm charts could include many more combinations of outcome risks (for example, for stroke and cancers), but we decided, for the sake of usability, to restrict the benefit-harm charts to stratification by sex, age, and 10-year risks of MI and severe GI bleeds. But even if not represented visually, all analyses included age- and sex-specific outcome risks for ischemic stroke, hemorrhagic stroke, the 12 cancers, and death, as explained above.

## Results

### Illustrations of the Benefit-Harm Charts

**Fig. 1:**
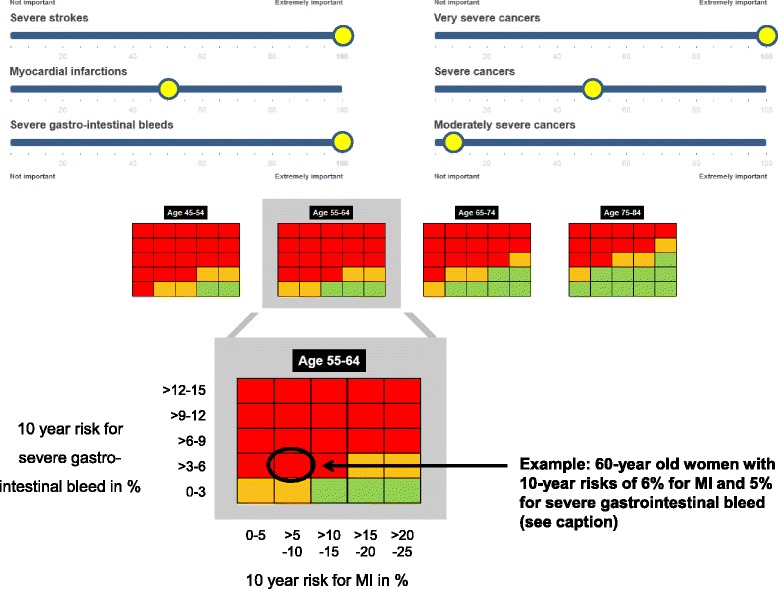
Benefit-Harm Chart for low dose aspirin on [http://www.benefit-harm-balance.com]. The Benefit-Harm Charts inform about the benefit-harm balance of low dose aspirin based on a patient’s risk profile. Benefit outcomes are (prevented) MI, severe ischemic stroke, and cancers, and harm outcomes are (excess) severe GI bleed and severe hemorrhagic stroke. In the first step the risk profile of an individual is determined using risk factors for the benefit and harm outcomes. Ideally, and to facilitate risk prediction, a computer processes the information on risk factors saved in the electronic patient chart and provides the 10-year risk estimates. In this example, the 60-year old woman has a 10-year risk of 6 % for MI, 6 % for severe ischemic stroke, 0.03 % for hemorrhagic stroke, 5 % for severe GI bleed (non-smoker, high blood pressure treated with atenolol tablet 50 mg per day, no diabetes, no history of gastric ulcer, no atrial fibrillation, no left-ventricular hypertrophy but chronic lower back pain; systolic blood pressure 145 mm Hg, total cholesterol 170 mg/dL, HDL cholesterol 55 mg/dL, takes ibuprofen tablet 200 mg twice a day. She eats vegetables regularly and is physically active. There is no prior history of colorectal cancer in her family). Using the online calculator [http://www.benefit-harm-balance.com] the Benefit-Harm Chart shows for specific outcome preferences (rulers on top) and for each combination of outcome risks (four 5x5 tables) the probability that aspirin provides more benefits than harms (red colored cells <40 % of the 100,000 repetitions with index >0, yellow colored cells 40–60 %, and green colored cells >60 % probability). For the 60-year old woman, the Benefit-Harm Chart shows that she is likely to experience more harms than benefits from aspirin

Figure 1 and [http://www.benefit-harm-balance.com] illustrate the Benefit-Harm Charts and explain how to use them using the example of a 60-year old woman with specific characteristics and preferences for the outcomes (see caption of Fig. 1). Based on risk prediction models that are calibrated for the region and population of the patient (for example, Caucasian, Boston area, USA), the 10-year risks for MI and severe ischemic stroke are elevated (6 % and 6 %, respectively). Her 10-year risk for severe GI bleed is slightly elevated (5 %) because she takes ibuprofen. The benefit-harm balance for this woman can be located (Fig. 1) by the respective sex, age, and risk categories for MI and severe GI bleeds. The red color indicates that the benefit-harm balance does not favor aspirin because the probability of overall benefit is low.

### Benefit-Harm Charts for women and men taking aspirin for primary prevention of CVD and cancer

**Fig. 2:**
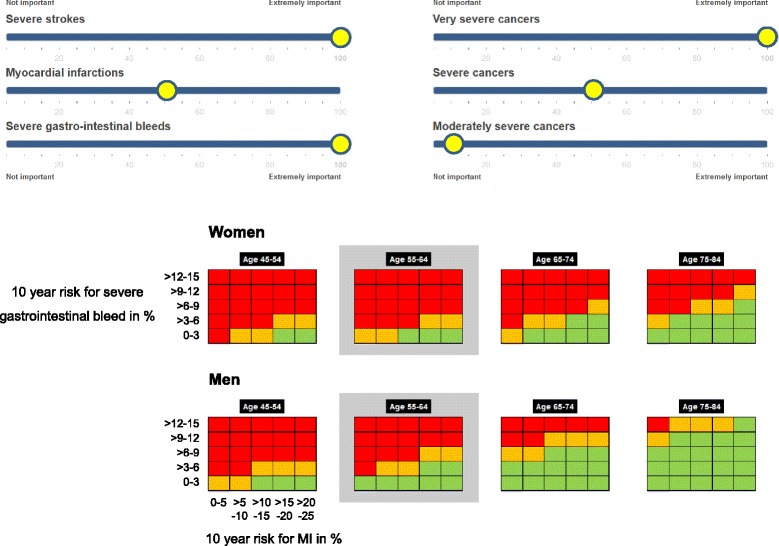
Benefit-Harm Chart for low dose aspirin for women and men. The Benefit-Harm Charts show the benefit-harm balance for four age categories and, within age categories, for 25 different combinations of 10-year risks for MI and severe GI bleeds. A comparison between women and men (for example, using age category 55–64 years) suggests that more men are likely to benefit from low dose aspirin than women. It is important to note that the Benefit-Harm Charts presented here focus on very low up to moderately high risks for MI (0–25 % 10-year risk) and severe GI bleeds (0–15 % 10-year risk). If the 10-year risks of severe GI bleeds are above 20 or 30 % (in elderly men and women who experienced gastric ulcers in the past [41]), the benefit-harm balance becomes unfavorable again since the number of excess severe GI bleeds under aspirin is high

The Benefit-Harm Chart in Fig. 2 illustrates how different outcome risks and their combinations influence the benefit-harm balance of low dose aspirin. The benefit-harm balance differs greatly across the primary prevention population. For example, there are many more men with different combinations of outcome risks who are likely to benefit from low dose aspirin than women, which is explained by their different risks for MI, stroke, and cancers. It is important to note that the Benefit-Harm Charts presented here focus on very low up to moderately high risks for MI (0–25 %) and severe GI bleeds (0–15 %), which covers a large proportion of a general population [40]. It does not include 10-year risks of severe GI bleeds above 15 %. If the 10-year risks of severe GI bleeds are above 20 or 30 % (in elderly men and women who experienced gastric ulcers in the past [41]), the benefit-harm balance becomes unfavorable again since the number of excess severe GI bleeds under aspirin is high. Within women and men, age is a strong determinant of the benefit-harm balance with more overall benefit as age increases). Also, the higher the risk of MI and the lower the risk for severe GI bleeds, the more likely is an overall benefit of aspirin.

**Fig. 3:**
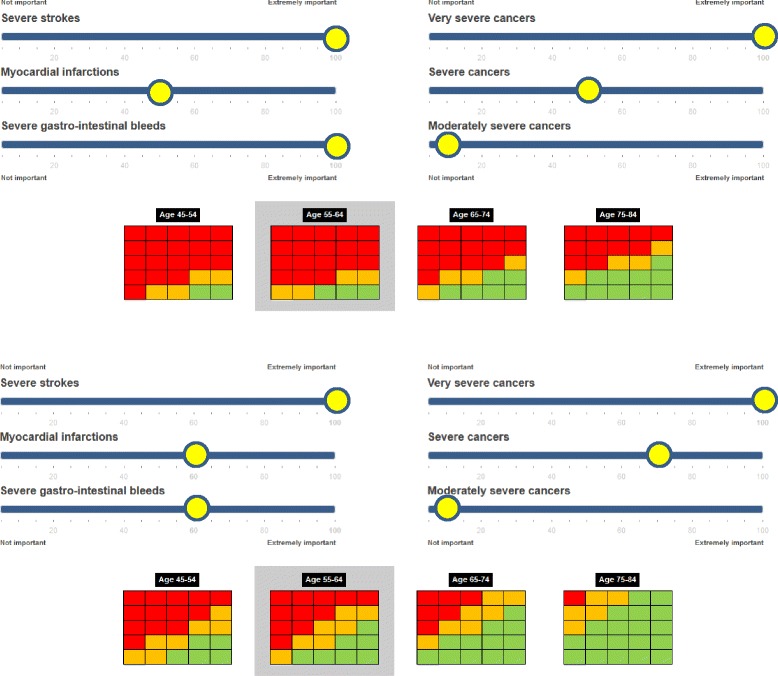
Impact of preferences on the benefit-harm balance. The two figures show the impact of putting different weights on the outcomes. In the upper Benefit-Harm Chart severe gastrointestinal bleeds are weighted substantially more than in the lower Benefit-Harm Chart. For more scenarios that reflect different preferences and outcome risks, refer to [http://www.benefit-harm-balance.com]

Figure 3 shows the impact of using different weights for the outcomes. The upper Benefit-Harm Chart shows the benefit-harm balances for women using weights that reflect the 5-year survival rates of the 16 outcomes (Table 2). Assume now that these women consider a severe stroke and cancers with 5-year survival rates <50 % (very severe cancers) to be the most important outcomes by far (100 on a scale from 0–100), followed by cancers with 5-year survival rate between 50 and 90 % (severe cancers, 70) and MI and severe GI bleeds (both 60), while cancers with 5-year survival rates >90 % (moderately severe cancers, 10) are considered less important. The lower Benefit-Harm Chart shows that low dose aspirin is likely to be beneficial for more individuals since less weight is put on severe GI bleeds. This example shows that preferences have a large impact on the benefit-harm balance of low dose aspirin. The online tool [http://www.benefit-harm-balance.com] offers the opportunity to assess how the benefit-harm balance changes according to individual preferences.

## Discussion

In this article, we conducted quantitative benefit-harm assessments of low dose aspirin for primary prevention of cardiovascular disease and cancer and we introduced our Benefit-Harm Charts that visualize the results for individuals. Our approach shows how two current limitations of guidelines on low dose aspirin, that is, separate recommendations for cardiovascular disease and cancer and population-based rather than more granulated recommendations, could be overcome. Besides the large impact of age and sex, the Benefit-Harm Charts show that individuals will derive a certain net benefit from aspirin depending not only on the risk for cardiovascular disease and severe GI bleeds but also on the importance attributed to different benefit and harm outcomes. The Benefit-Harm Charts may support policy and guideline developers and also regulatory agencies in making more finely granulated recommendations and decisions. In addition, the Benefit-Harm Charts can inform questions across diseases that share a common preventive or therapeutic intervention. Patients and physicians may be able to make individualized treatment decisions based on combinations of outcome risks and preferences.

### Going beyond the simple approaches for benefit-harm assessment

Estimating the benefit-harm balance is challenging for many health care decisions because so many factors need to be considered. Currently physicians and individuals have to either simplify the task and use approaches like the numberneeded totreat or number needed to harm or not use a quantitative approach to assess benefit-harm balance at all. Table 1 shows how guidelines result in different recommendations when using a simplified or no approach to estimate the benefit-harm balance. In contrast, comprehensive quantitative benefit-harm assessments that consider all relevant factors that have an impact on the benefit-harm balance allow for testing the impact of different assumptions (such as preferences) on the benefit-harm balance. To bridge the gap between population-based estimates of the benefit-harm balance (that focus on very few and averaged combinations of outcome risks) and individual preventive counseling, quantitative benefit-harm assessments can be repeated thousands of times for any possible risk profile and preferences for outcomes, and the results can be visualized through Benefit-Harm Charts. Based on Benefit-Harm Charts it becomes easier to issue more nuanced recommendations that reduce over- and underuse of interventions. Such an approach could address the current lack of systematic and transparent approaches to estimate the balance of benefits and harms during the development of many guidelines and reduce disagreement among guidelines [12].

However, some simplifications are currently necessary when Benefit-Harm Charts are developed. For example, we repeated the benefit-harm analyses “just” for women and men and for four age categories and 25 combinations of outcome risks for MI and GI bleeds (thus 2*4*25 = 200 different profiles). Thus we simplified this second step by not repeating the analyses for any combination of the 16 outcome risks but just for combinations of sex, age, and outcome risks for MI and severe GI bleeds, all of which have a substantial impact on the benefit-harm balance of aspirin. For a visual and real-time display (as on [http://www.benefit-harm-balance.com]) such simplifications are (currently) necessary. One could also produce more individualized Benefit-Harm Charts where different outcome risks are considered for all outcomes or where the benefit-harm balance would be calculated even for one individual. It is challenging to make the simulations so fast that the user can obtain a real-time estimate of his or her (fully) individual benefit-harm balance.

### Moving guidelines forward

The Benefit-Harm Charts show that a substantial proportion of the population is likely to benefit from low dose aspirin for primary prevention of cardiovascular disease and cancer. For example, a large proportion of the general population has a low 10-year risk (<3 %) for severe GI bleeds [40, 41]. Assuming the weights used for Fig. 2, men of all four age classes and at 10-year risks for severe GI bleeds of <10 % (no previous gastric ulcer, no nonsteroidal anti-inflammatory agents [41]) are likely to benefit from aspirin irrespective of their risk of MI. Elderly women with at least moderately increased risk of MI are also likely to benefit from aspirin. Even when assuming that the proportion of men and women changes if outcomes are weighted differently, a substantial proportion of men and women are likely to benefit.

The Benefit-Harm Charts show the limitations of current guidelines: The recommendations are not granular enough to accommodate the great variation of the benefit-harm balance of aspirin across the population. Guidelines use few parameters to guide individualized prevention and do not base their recommendations on quantitative benefit-harm assessments except for the USPSTF. As a consequence, there is a risk for under- and overtreatment. Also current guidelines are likely to underestimate the overall benefit from aspirin because they focus on cardiovascular disease and cancer separately. Thereby, the same harms are counted twice (in the cardiovascular and in the cancer guidelines against prevented outcomes). The benefit-harm analyses and the Benefit-Harm Charts presented here thus support the idea of combining outcomes across disease areas if they are prevented or treated by the same intervention [22].

### Research needs

Although the Benefit-Harm Charts may appear attractive, substantial research is still needed to bring them to practice. For example, to consider the importance individuals assign to outcomes, preference-eliciting tools need to be developed that are easily understood by individuals and implementable. Standard methods such as standard gamble or time trade-off are complicated for respondents and unrealistic for use in a practice setting [42]. Best-worst scaling, which requires individuals to repeatedly choose one outcome over another, pictorial representations of illness, or some more informal techniques are likely to be preferable [43–45]. In addition, the presentation format of the Benefit-Harm Charts needs to be tested using qualitative and quantitative research [46]. Also, the impact of using Benefit-Harm Charts on the decision-making process must be assessed through trials, as is common for decision aids [47]. Finally, outcome risks, evidence on treatment effects, and preferences may change over time. For example, there are ongoing studies (ASPREE, ARRIVE, ASCEND, ACCEPT-D) that assess the effects of aspirin in diverse settings and populations as well as cost effectiveness [48, 49]. Technically, it is not difficult to update benefit-harm analyses such as the ones presented here with additional evidence. But an important question is how to organize such updates efficiently so that decision makers can always rely on up-to-date Benefit-Harm Charts. The use of modern information and computing technology may prove valuable to support an update process where, for example based on a Bayesian framework, the additional evidence adds to existing benefit-harm assessments and Benefit-Harm Charts.

### Strengths and limitations of study

We did not perform extensive sensitivity analyses as commonly reported for quantitative benefit-harm analyses [24, 25, 50, 51] but provided an example for how the choice of weights impacts the benefit-harm balance (Fig. 2). The Gail/NCI approach can easily accommodate such sensitivity analyses that consider different weights [http://www.benefit-harm-balance.com], heterogeneity of treatment effects, or a greater number of outcomes. Such sensitivity analyses could also consider recent insights into the time-varying effects of aspirin, which may not show beneficial effects until at least 3 years after the start of use, but whose benefits may be sustained for several years even after cessation in long-term users [52]. Second, we assumed a simplified decision-making context, where we did not consider additional conditions, (for example, chronic lung disease), treatments (such as proton pump inhibitors), or events during the 10 years of prevention (for example, gastrointestinal bleed) that may influence the risk profile or the propensity of taking aspirin. Finally, we refrained from making recommendations about which prediction models to use and from making recommendations for or against low dose aspirin, since the necessary judgments needed for developing recommendations in a guideline depend on more factors than the ones we considered here [8, 9].

## Conclusion

Extensive quantitative benefit-harm analyses for many different combinations of outcome risks go well beyond benefit-harm assessments made by policy makers, regulators, or guideline developers that are often made for entire populations only and not based on quantitative benefit-harm analyses. The example of low dose aspirin for primary prevention of cardiovascular disease and cancer shows that it may be time for guideline developers to provide combined recommendations for different diseases that may be prevented or treated by the same intervention. Visualization of results by Benefit-Harm Charts provides a novel way to make more finely granulated policy decisions and recommendations but also to inform individualized decisions on interventions. This approach may avoid some of the simplifications made by current regulatory process and guidelines, which carry greater risk of over- and undertreatment.

## References

[1] National Heart, Lung and Blood Institute. Risk Assessment Tool for Estimating Your 10-year Risk of Having a Heart Attack. http://cvdrisk.nhlbi.nih.gov. Accessed 27 Sept 2015.

[2] Framingham Heart Study (1994). Stroke risk calculator. Based on D’Agostino et al. Stroke Risk Profile: Adjustment for Antihypertensive Medication. Stroke.

[3] National Cancer Institute. Colorectal Cancer Risk Assessment Tool. http://www.cancer.gov/colorectalcancerrisk/. Accessed 27 Sept 2015.

[4] Mendis S, Puska P, Norrving B. WHO | Global atlas on cardiovascular disease prevention and control. Geneva: World Health Organization; 2011. http://www.who.int/cardiovascular_diseases/publications/atlas_cvd/en/. Accessed 27 Sept 2015.

[5] Perk J, De Backer G, Gohlke H, Graham I, Reiner Z, Verschuren WM (2012). European Guidelines on cardiovascular disease prevention in clinical practice. The Fifth Joint Task Force of the European Society of Cardiology and Other Societies on Cardiovascular Disease Prevention in Clinical Practice. Eur Heart J.

[6] United States Preventive Services Task Force (2009). Aspirin for the Prevention of Cardiovascular Disease: U.S. Preventive Services Task Force Recommendation Statement. Ann Intern Med.

[7] United States Preventive Services Task Force (2007). Routine aspirin or nonsteroidal anti-inflammatory drugs for the primary prevention of colorectal cancer: U.S. Preventive Services Task Force recommendation statement. Ann Intern Med.

[8] Guyatt GH, Oxman AD, Vist GE, Kunz R, Falck-Ytter Y, Alonso-Coello P (2008). GRADE: an emerging consensus on rating quality of evidence and strength of recommendations. BMJ..

[9] Guyatt GH, Oxman AD, Kunz R, Falck-Ytter Y, Vist GE, Liberati A (2008). Going from evidence to recommendations. BMJ..

[10] Vandvik PO, Lincoff AM, Gore JM, Gutterman DD, Sonnenberg FA, Alonso-Coello P (2012). Primary and secondary prevention of cardiovascular disease: Antithrombotic Therapy and Prevention of Thrombosis, 9th ed: American College of Chest Physicians Evidence-Based Clinical Practice Guidelines. Chest..

[11] JBS3 Board (2014). Joint British Societies’ consensus recommendations for the prevention of cardiovascular disease (JBS3). Heart.

[12] Yu T, Vollenweider D, Varadhan R, Li T, Boyd C, Puhan MA (2013). Support of personalized medicine through risk-stratified treatment recommendations - an environmental scan of clinical practice guidelines. BMC Med..

[13] Habbema JDF, Wilt TJ, Etzioni R, Nelson HD, Schechter CB, Lawrence WF (2014). Models in the development of clinical practice guidelines. Ann Intern Med..

[14] Berger JS, Lala A, Krantz MJ, Baker GS, Hiatt WR (2011). Aspirin for the prevention of cardiovascular events in patients without clinical cardiovascular disease: a meta-analysis of randomized trials. Am Heart J.

[15] Man-Son-Hing M, Laupacis A, O’Connor AM, Coyle D, Berquist R, McAlister F (2000). Patient preference-based treatment thresholds and recommendations: a comparison of decision-analytic modeling with the probability-tradeoff technique. Med Decis Making..

[16] Fried TR, Tinetti ME, Towle V, O’Leary JR, Iannone L (2011). Effects of benefits and harms on older persons’ willingness to take medication for primary cardiovascular prevention. Arch Intern Med..

[17] Gail MH, Costantino JP, Bryant J, Croyle R, Freedman L, Helzlsouer K (1999). Weighing the risks and benefits of tamoxifen. J Natl Cancer Inst..

[18] Greenhalgh T, Howick J, Maskrey N (2014). Evidence based medicine: a movement in crisis?. BMJ..

[19] Graham ID, Tetroe J (2007). Some theoretical underpinnings of knowledge translation. Acad Emerg Med..

[20] Pronovost PJ (2013). Enhancing physicians’ use of clinical guidelines. JAMA..

[21] Aspirin for the Prevention of Cardiovascular Disease: Preventive Medication. http://www.uspreventiveservicestaskforce.org/BrowseRec/Search?s=aspirin. Accessed 27 Sept 2015.

[22] Thun MJ, Jacobs EJ, Patrono C (2012). The role of aspirin in cancer prevention. Nat Rev Clin Oncol..

[23] Puhan MA, Singh S, Weiss CO, Varadhan R, Boyd CM (2012). A framework for organizing and selecting quantitative approaches for benefit-harm assessment. BMC Med Res Methodol..

[24] Boyd C, Singh S, Varadhan R, Weiss CO, Sharma R, Bass EB (2012). Methods for benefit and harm assessment in systematic reviews. Agency for Healthcare Research and Quality.

[25] Stegeman I, Bossuyt PM, Yu T, Boyd C, Puhan MA (2015). Aspirin for primary prevention of cardiovascular disease and cancer. A benefit and harm analysis. PLoS One..

[26] Puhan MA, Singh S, Weiss CO, Varadhan R, Sharma R, Boyd CM (2013). Evaluation of the benefits and harms of aspirin for primary prevention of cardiovascular events. Agency for Healthcare Research and Quality.

[27] Berger JS, Roncaglioni MC, Avanzini F, Pangrazzi I, Tognoni G, Brown DL (2006). Aspirin for the primary prevention of cardiovascular events in women and men: a sex-specific meta-analysis of randomized controlled trials. JAMA..

[28] Algra AM, Rothwell PM (2012). Effects of regular aspirin on long-term cancer incidence and metastasis: a systematic comparison of evidence from observational studies versus randomised trials. Lancet Oncol..

[29] NHLBI Incidence and Prevalence Chartbook. https://www.nhlbi.nih.gov/research/reports/2006-incidence-chart-book.htm. Accessed 27 Sept 2015.

[30] Sacco S, Marini C, Toni D, Olivieri L, Carolei A (2009). Incidence and 10-year survival of intracerebral hemorrhage in a population-based registry. Stroke..

[31] U.S. Cancer Statistics Working Group. United States Cancer Statistics: 1999–2012 Incidence and Mortality Web-based Report. Atlanta: U.S. Department of Health and Human Services, Centers for Disease Control and Prevention and National Cancer Institute; 2014. www.cdc.gov/uscs. Accessed 27 Sept 2015.

[32] Centers for Disease Control and Prevention. Deaths: Final Data for 2013. National Vital Statistics Reports. Volume 64, Number 2. http://www.cdc.gov/nchs/deaths.htm. Accessed 13 July 2015.

[33] Cost Effectiveness Analysis Registry. Tufts Medical Center, USA, www.cearegistry.org. Accessed 27 Sept 2015.

[34] Bata IR, Gregor RD, Wolf HK, Brownell B (2006). Trends in five-year survival of patients discharged after acute myocardial infarction. Can J Cardiol..

[35] Hankey GJ, Jamrozik K, Broadhurst RJ, Forbes S, Burvill PW, Anderson CS (2000). Five-year survival after first-ever stroke and related prognostic factors in the Perth Community Stroke Study. Stroke..

[36] Roberts SE, Button LA, Williams JG (2012). Prognosis following upper gastrointestinal bleeding. PLoS One..

[37] Howlader N, Noone AM, Krapcho M, Garshell J, Miller D, Altekruse SF, et al. SEER Cancer Statistics Review, 1975–2012. National Cancer Institute. Bethesda, MD, http://seer.cancer.gov/csr/1975_2012/. Accessed 27 Sept 2015.

[38] Minelli C, Abrams KR, Sutton AJ, Cooper NJ (2004). Benefits and harms associated with hormone replacement therapy: clinical decision analysis. BMJ..

[39] R Foundation for Statistical Computing. R: A language and environment for statistical computing. 2014. http://www.r-project.org/. Accessed 27 Sept 2015.

[40] Thorat MA, Cuzick J (2015). Prophylactic use of aspirin: systematic review of harms and approaches to mitigation in the general population. Eur J Epidemiol..

[41] Hernández-Díaz S, García Rodríguez LA (2006). Cardioprotective aspirin users and their excess risk of upper gastrointestinal complications. BMC Med..

[42] Puhan MA, Guyatt GH, Montori VM, Bhandari M, Devereaux PJ, Griffith L (2005). The standard gamble demonstrated lower reliability than the feeling thermometer. J Clin Epidemiol..

[43] Brett Hauber A, Fairchild AO, Reed JF (2013). Quantifying benefit-risk preferences for medical interventions: an overview of a growing empirical literature. Appl Health Econ Health Policy..

[44] Flynn TN (2010). Valuing citizen and patient preferences in health: recent developments in three types of best-worst scaling. Expert Rev Pharmacoecon Outcomes Res..

[45] Büchi S, Sensky T, Sharpe L, Timberlake N (1998). Graphic representation of illness: a novel method of measuring patients’ perceptions of the impact of illness. Psychother Psychosom..

[46] Zipkin DA, Umscheid CA, Keating NL, Allen E, Aung K, Beyth R (2014). Evidence-based risk communication: a systematic review. Ann Intern Med..

[47] Stacey D, Légaré F, Col NF, Bennett CL, Barry MJ, Eden KB (2014). Decision aids for people facing health treatment or screening decisions. Cochrane Database Syst Rev..

[48] Ademi Z, Liew D, Hollingsworth B, Steg PG, Bhatt DL, Reid CM (2013). Is it cost-effective to increase aspirin use in outpatient settings for primary or secondary prevention? Simulation data from the REACH Registry Australian Cohort. Cardiovasc Ther..

[49] Clinical Trials Gov. https://clinicaltrials.gov/ct2/search. Accessed 27 Sept 2015.

[50] Yu T, Fain K, Boyd CM, Singh S, Weiss CO, Li T (2014). Benefits and harms of roflumilast in moderate to severe COPD. Thorax..

[51] Mt-Isa S, Hallgreen CE, Wang N, Callréus T, Genov G, Hirsch I (2014). Balancing benefit and risk of medicines: a systematic review and classification of available methodologies. Pharmacoepidemiol Drug Saf..

[52] Cuzick J, Thorat MA, Bosetti C, Brown PH, Burn J, Cook NR (2015). Estimates of benefits and harms of prophylactic use of aspirin in the general population. Ann Oncol..

